# Exploring men's and women's experiences of depression and engagement with health professionals: more similarities than differences? A qualitative interview study

**DOI:** 10.1186/1471-2296-8-43

**Published:** 2007-07-24

**Authors:** Carol Emslie, Damien Ridge, Sue Ziebland, Kate Hunt

**Affiliations:** 1 MRC Social & Public Health Sciences Unit, Glasgow, UK; 2 School of Integrated Health, Westminster University, London, UK; 3 Department of Primary Health Care, Oxford University, Oxford, UK

## Abstract

**Background:**

It is argued that the ways in which women express emotional distress mean that they are more likely to be diagnosed with depression, while men's relative lack of articulacy means their depression is hidden. This may have consequences for communicating with health professionals. The purpose of this analysis was to explore how men and women with depression articulate their emotional distress, and examine whether there are gender differences or similarities in the strategies that respondents found useful when engaging with health professionals.

**Methods:**

In-depth qualitative interviews with 22 women and 16 men in the UK who identified themselves as having had depression, recruited through general practitioners, psychiatrists and support groups.

**Results:**

We found gender similarities and gender differences in our sample. Both men and women found it difficult to recognise and articulate mental health problems and this had consequences for their ability to communicate with health professionals. Key gender differences noted were that men tended to value skills which helped them to talk while women valued listening skills in health professionals, and that men emphasised the importance of getting practical results from talking therapies in their narratives, as opposed to other forms of therapy which they conceptualised as 'just talking'. We also found diversity among women and among men; some respondents valued a close personal relationship with health professionals, while others felt that this personal relationship was a barrier to communication and preferred 'talking to a stranger'.

**Conclusion:**

Our findings suggest that there is not a straightforward relationship between gender and engagement with health professionals for people with depression. Health professionals need to be sensitive to patients who have difficulties in expressing emotional distress and critical of gender stereotypes which suggest that women invariably find it easy to express emotional distress and men invariably find it difficult. In addition it is important to recognise that, for a minority of patients, a personal relationship with health professionals can act as a barrier to the disclosure of emotional distress.

## Background

It is consistently reported that women have higher rates of depression than men. In the past, there was an assumption that women were more vulnerable to depression. However, higher rates of suicide for men than women in almost every country, and rising rates of depression for men in many countries, has led to the development of more sophisticated approaches to conceptualising gender differences in mental health [1,2]. One approach focuses on the importance of socially constructed gender roles; in many societies, culturally dominant forms of masculinity emphasise strength, stoicism and emotional control, in direct contrast with femininity which is characterised by vulnerability and emotional expression [3,4]. Thus, it is hypothesised that the ways that women express emotional distress mean that they are more likely to be diagnosed (and perhaps over-diagnosed) with depression, while men's lack of articulacy means their depression may be 'hidden' [5,6].

Qualitative studies are necessary in order to explore how people perceive, construct and express depression (see, for example, [7-12]). Qualitative researchers have also sought to explore how people with mental health problems experience primary care. Mental health patients valued general practitioners (GPs) who knew them, listened to them and took their concerns seriously [13,14]. The importance of continuity of care has been highlighted as patients have suggested that a long-standing relationship with a GP enabled trust to develop, prevented them having to repeatedly retell their stories and, perhaps most importantly, allowed them and health professionals to understand each other as people [14,15]. Symptoms of depression also contribute to problems in accessing help; for example, lack of confidence and assertiveness, low self-worth and feelings of lack of entitlement to GP time and resources have deterred patients from raising concerns about emotional distress [10,13,14,16]. Another frequently reported barrier to communication in primary care is that patients are uncertain about the nature of their emotional distress and lack the vocabulary with which to name and discuss their experiences [8-10,13]. Karp [7] describes this period of 'inchoate feelings' as the beginning of the depression 'career' and argues that "part of the pain of depression is their (people with depression) inability to satisfactorily communicate what they are feeling, and the simultaneous failure of others to understand them" (p27). Even when patients had the vocabulary with which to explain their emotional distress, they were sometimes unsure whether their symptoms could be classified as forms of medically manageable illness which were suitable for discussion with a GP [10,17]. Pollock [18] found that patients suppressed the expression of emotional distress during medical consultations because they were trying to 'maintain face' and conform to "the socially sanctioned role of the stoic, good and uncomplaining patient in order to retain the social esteem and good will of others" (p175).

Despite this increasing body of qualitative work, very few studies have compared the experiences of men and women. There is still relatively little discussion of men's mental health [19]; many studies concentrate solely on women (e.g. [20-23]), and those with mixed samples tend to include a much higher proportion of women than men (e.g. [10,12,16,17]). Some studies do comment, in passing, on gender differences but do not explicitly set out to compare the experiences of men and women. For example, Rogers and colleagues [10] note that, while most of their respondents were reluctant to disclose to their doctor that they felt they couldn't cope, this reluctance was particularly evident in the accounts of the men, while Wittink and colleagues [17] found that only women in their sample expressed a belief that doctors were able to 'pick up' on depression without the patient having to be explicit about their emotions. Karp [7] has argued that future research should concentrate on how gender and other markers of social position influence meanings attached to depression, so that "proper respect can be accorded to both regularities in the illness experience and to the diversity of definitions that affect its course" (p27).

Two recent studies have explicitly compared men and women. They concluded that men and women experience depression in a similar way, but have different ways of manifesting and expressing their distress. Brownhill and colleagues [5] found that both men and women tried to avoid emotional distress by not thinking about problems, or by distancing and distracting themselves. Men were more likely to suppress emotions which then built up over time and were sometimes released in the form of anger or violence, while women were more prepared to release emotions early by crying and seeking help. However, this study used a convenience sample of teachers and students who discussed their experiences of being 'down in the dumps'. In contrast, Danielsson and Johansson [24] used a sample of people diagnosed with depression. They found that women verbalized emotional distress more readily than men (which may put them at risk of being over-diagnosed with depression), and men talked more easily about physical distress than about emotions (which may put them at risk of being under-diagnosed for depression). The authors concluded that men might 'retreat into silence' when faced with emotional distress and so clinicians need to listen to the 'avoided' and the 'unarticulated' (p176), rather than just what is expressed in words, in order to counteract stereotypes around gender and depression.

In this paper, we take a gender comparative [25] approach to exploring the mental health of men and women. We are aware that language in this area is problematic. Oakley [26] popularised the influential distinction between sex and gender in the 1970s, where 'sex' referred to biological differences between males and females (assumed to be universal and unchanging), and 'gender' referred to culturally constructed notions of masculinity and femininity (assumed to be highly variable). While this distinction has been useful theoretically, it is increasingly recognised as problematic (see Emslie et al [27] for a full discussion). For example, the biologically 'given' nature of sex has been shown to be more complex than previously recognised [28]. It has also been argued that there is constant interaction between the biological and the social; biological 'facts' are socially constructed, while cultural views affect which 'facts' are selected and how much importance they are given [29,30].

Recently, Annandale [31] has argued that we need to move towards a new system: "in which social (gender) and biological (sex) depend on each other for understanding just as much as before, but where the meaning of biological sex and the meaning of social gender, as well as the connections between them, are more fluid" (p88). Some might argue that in this analysis we are taking a 'sex comparative' approach as we compare groups of (biologically defined) men with groups of (biologically defined) women. However, we have chosen to use the term 'gender' as the term 'sex' still carries connotations of narrow biological differences and we wish to indicate the importance of socially constructed gender roles (while not denying the role that biology plays in mental health). Furthermore, we believe that experiences of talking about mental health problems would seldom be heard in a 'gender-free' way as the listener is likely, consciously or unconsciously, to interpret what is being said to them in the light of the speaker's sex and socially constructed scripts of gender appropriate behaviour.

Here, gender is conceptualised as a dynamic set of socially constructed relationships, rather than as a fixed and binary category [32]. Conceptualising gender in this way disrupts the notion that 'masculine' and 'feminine' identities are stable characteristics of individuals, and problematises the notion of focusing exclusively on gender difference. Instead, we are also interested in exploring commonalities across men and women, and diversity among women and among men.

In this paper, we present data from in-depth interviews with 38 respondents who identified themselves as having had depression. In our analysis, we set out to compare the accounts of men and women: specifically, is there evidence that women are articulate about depression and men are inarticulate, and are there gender differences or similarities in the strategies which respondents described as useful in helping them to engage with health professionals?

## Methods

### The DIPEx depression study

This paper describes a secondary analysis of in-depth narrative interviews. The interviews were originally collected for the depression module of the DIPEx (personal experiences of health and illness) website [33,34]. This website is unique because the experiences presented are based on interviews collected and analysed by experienced qualitative researchers using rigorous methods approved by the UK Multi-centre Research Ethics Committee. The website features short extracts of people's experiences but the full in-depth interviews are available for secondary analysis (see for example [35,36]).

Following the standard requirements for DIPEx modules, interviews were conducted with a maximum variation sample [37] of people with experience of depression. Participants were recruited to include men and women from various age groups, ethnic and social class backgrounds, and locations in the UK. In 2003 and 2004, 38 people (16 men and 22 women: see table 1) were interviewed for the depression module – usually in their own home – by a male social scientist (DR) with extensive experience of interviewing on sensitive topics. Respondents had to be over the age of 18, identify as having had depression, and be feeling sufficiently well to undertake the interview. People were invited to take part through GPs, psychiatrists, support groups and newsletters. Most (34) of the 38 respondents had experienced multiple or prolonged episodes of depression and around half (18) had been hospitalised for depression or mania.

**Table 1:** Characteristics of respondents

	(N = 38)
**Age at interview**	

<30	3
30–40	14
41–55	11
56–65	6
66+	4

**Ethnicity**	

White British	33
Black	1
Asian	1
South European	1
North European	1
American	1

**Main type of depression reported during interview**	

Unipolar	28
Bipolar	10

**'Most helpful' treatments reported during interview **(many respondents reported more than one treatment)	

Talking therapies	31
Medication	24
Holistic approach	9
Doctor support	8
Hospitalisation	5
Electroconvulsive therapy (ECT)	1

Open-ended, in-depth interviews were conducted in which respondents were able to focus on issues that were important to them. First, they were asked to tell their own story of developing depression, with little interruption. Subsequently, DR used questions and prompts to ensure that particular issues were explored (e.g. life before depression; the period of time when things seemed 'not quite right'; the depression experience(s); social consequences of depression; help seeking and personal coping strategies). Interviews ranged from 90 minutes to 180 minutes and were recorded by audio and/or video with the consent of respondents. Audio recordings were professionally transcribed and transcripts were returned to respondents for checking.

### Analysis

Analysis was undertaken in two phases. DR and SZ conducted the first phase of analysis. DR identified emerging themes based on a 'modified grounded theory' approach [38], produced summary tables of the key features of interviews, wrote thematic topic summaries which feature on the website, some of which touch on gender (see "talking about.." sections on 'recognition and diagnosis', 'doctors', 'psychiatrists and other professionals', and 'talking therapies' [39], and developed papers on the overall analysis which include difficulties in recognising depression and relating to professionals [9]. This initial analysis was clarified and scrutinised by SZ and DR through regular meetings and electronic exchanges.

In the second phase of analysis, CE undertook secondary analysis of the data in order to address a new research question: is there evidence that women are articulate about depression and men are inarticulate, and are there gender differences or similarities in the strategies which respondents described as useful in helping them to engage with health professionals? (A previous secondary analysis by the same team on a subset of these data explored the variety of strategies men with depression used to reconstruct a valued sense of themselves and their own masculinities [35]).

There has been debate about the secondary analysis of qualitative data; while some argue that the researcher who collects the data is the person best placed to undertake analysis, others assert that the 'distance' an independent researcher has from the data can be helpful [40]. In this case, the 'gap' between primary and secondary researchers was bridged through discussion about the context of the study and the data collection process [40]. In order to aid familiarization, all the transcripts were read repeatedly and the raw data were recoded in accordance with the new research question.

Following McCracken [41], analysis moved from the particular (a detailed analysis of language in each transcript) to the general (a comparison of patterns and themes across all the transcripts). Thematic analysis was used to identify recurring patterns in the data, with due attention paid to any deviant cases [42]. QSR Nvivo 2.0 [43] was used to facilitate the analysis of themes and explore (our interpretation of) the underlying reasoning of respondents. Emerging themes were identified, tested against the transcripts, and where necessary re-formulated in a cyclical process. All authors were involved in debating and refining the final interpretation of the data and commenting on a number of drafts of the paper.

## Results

### Overview

Given our gender comparative approach to these data, we first report similarities between men and women, and then report differences. First, both men and women found it difficult to recognise and articulate mental health problems and this had consequences for their ability to communicate with health professionals. Men and women reported diverse relationships with health professionals; some valued a close personal relationship with health professionals, while others felt that this personal relationship was a barrier to communication and preferred 'talking to a stranger'. Secondly, we identified some gender differences in these data. While both men and women valued good communication skills in health professionals, men tended to value skills which helped them to talk, while women valued listening skills. In addition, men tended to emphasise the importance of getting practical results from talking therapies in their narratives, as opposed to other forms of therapy which they conceptualised as 'just talking'. We present extracts from respondents' narratives in order to illustrate these themes. (For each quotation, we give the respondent's ID number followed by 'w' to indicate that the speaker is a woman or 'm' to indicate the speaker is a man).

### Similarities in the accounts of men and women with depression

#### 1) DIFFICULTIES IN RECOGNISING AND ARTICULATING MENTAL HEALTH PROBLEMS

Both men and women were willing to talk in some depth about their feelings and experiences of depression in the interviews. However, it was universally difficult for people to find the words to describe their experience of depression, at least initially. Both men and women discussed how they lacked the appropriate vocabulary to describe how they were feeling, both to themselves and to others (see also [9]):-

*DP01m: I felt so utterly lonely.. and I couldn't reach out to explain to people*.

*DP38m: I didn't have a name for depression, I didn't know what it was*.

*DP28w: It was terrifying .. I couldn't get across to people how I was feeling*.

*DP35w: I had no lexicon, I had no desire to communicate*.

To begin with, many respondents had doubts about the value of therapies which involved talking as a means of recovery. However, there was a recognition that not wanting to talk about what was wrong, or putting a 'brave face' on it, could be part of the experience of depression. Indeed, respondents noted that, ironically, the time they most needed help was when they were least capable of asking for it (see also [21,44] for similar findings).

Such difficulties in putting words to experiences had consequences when respondents tried to seek professional help, particularly when consulting general practitioners who are often the first point of contact for people with depression in the UK. For example, DP02w described how it had taken her years to understand what she was being asked, and what she was expected to say, when consulting a GP, while DP26w emphasised the importance of being able to articulate her experiences of depression when consulting a series of different GPs who were not aware of her history :-

*DP02w: It's taken me years to find out what the.. official symptoms are of depression. Now if I go to the doctor's I know what they're asking me, and I know what they're expecting me to say*.

*DP26w: Every time you go back to your surgery, you see a different GP.. you just have to prepare yourself that it's OK to feel like this, I'm not being a fraud.. .it's really, really hard because if you are depressed anyway you have got such low self esteem that you feel a complete fool.. it is about the way you present as well and the way you can articulate yourself*.

DP05m found it particularly difficult to seek help as he had no desire to talk to anyone, let alone a health professional. Eventually, his wife persuaded him to go to his GP, but he continued to find it very hard to describe his feelings:-

*DP05m: I didn't want to talk to anybody .. even if I had wanted to I couldn't have made the effort to actually go out and initiate a contact with .. a health professional .(After being persuaded to see GP).. I couldn't articulate how I was feeling. ..Ask me a direct question. "Are you feeling suicidal?" "Yes".. I could answer ..but sort of saying.. "How do you feel?" I wasn't capable of articulating that*.

#### 2) VALUED FEATURES OF RELATIONSHIPS WITH HEALTH PROFESSIONALS

In consultations with health professionals, both men and women valued being listened to, being taken seriously, not being rushed and talking to someone who was sympathetic and caring and who trusted them. Both men and women discussed the need to have some 'rapport' (DP36m), 'connection' (DP38w) or to 'click' (DP10m) or 'gel' (DP27w) with their GP and those who provided talking therapies. However, respondents in the sample differed as to the type of relationship they found most helpful in facilitating communication. These differences did not simply fall along gendered lines; instead, there was diversity in the preferences of both men and women.

##### a) Close personal relationships with health professionals

Some men and women emphasised how much they appreciated a close relationship with health professionals. Continuity of care was valued both in terms of building a personal relationship and because of the difficulty of continually having to repeat one's story to different health professionals. DP01m referred to his GP of 15 years as a 'friend', while two other men (DP10m and DP16m) had developed friendships with their counsellors:-

*DP01m: My GP .. is an extraordinarily kind man. He helped me care for my wife (when she was dying) and so we've a great understanding and he's a friend more than anything else*.

*DP10m: I just clicked with him (counsellor).. He was.. a very, very warm person.. very non judgemental. And I could talk about anything completely openly*.

*DP16m: A very special man in many ways (counsellor)..he became, and still is, a personal friend*.

Women described some of their relationships with health professionals in even more personal terms, putting strong emphasis on their human, as well as their professional, qualities. DP15w described how her female GP had hugged her to encourage her to have counselling for her suicidal thoughts while DP27w described how 'there was a hug there (from the nurses) when you needed it' when she was hospitalised after a suicide attempt. Some women used language more often associated with personal than professional relationships when discussing their therapists. For example, DP22w felt that her relationship with her therapist was "probably one of the longest, most meaningful relationships in my life" while DP20w compared the therapeutic relationship to marriage:-

*DP20w: I guess it's like marriage, you've gotta kind of work with someone initially to sort of figure out whether or not you can work with them because you're gonna be seeing them for a while*.

##### b) Talking to a 'stranger' and preserving a degree of anonymity

In contrast, other respondents felt that an existing relationship with a health professional could act as a barrier to communication and so preferred to talk about depression with someone they did not know in another context. For example, DP06w and DP05m explained how having a friendship with their GP made it very difficult to discuss depression, while DP07m felt he did not want his longstanding psychoanalyst to see him in a deep depression, perhaps because of his pride:-

*DP06w: (I was).. working with my GP on a professional basis (as a nurse). (It) made it difficult because he was also a good friend We saw each other socially.. I felt that if he knew the real me, he wouldn't necessarily want to go out and spend social time.. it was like uncovering something in myself which at that time I saw as a weakness*.

*DP05m: It's actually a lot easier to talk to strangers about depression than it is to talk to people that you know and I consider my GP as essentially as a friend. (I) found it really very, very difficult to, to fully talk about how I felt, and that I tried to kill myself*.

*DP07m: I remember I had one depression for seven months.. and I made a conscious decision of not seeing my psychoanalyst.. it was almost as if I didn't want him to see me as bad as I was.. I wouldn't say I was ashamed, but it's almost like some kind of pride*.

Similarly, three other respondents felt that some sort of distance between themselves and their therapists helped the process of communication. DP04m found it easier to talk because he was paying 'some poor sod', DP27w found it easier to open up to someone who doesn't 'know me from Adam' while DP34w emphasised the benefit of talking to 'a complete stranger':-

*DP04m: It was nice to be paying somebody (counsellor) a lot of money to talk.. you talk about yourself, and you can't bend someone's ear all day about it, but you need to. It wouldn't be fair to anyone else. You're paying some poor sod who has to listen to it*.

*DP27w: It was a relief being able to talk about things that I'd never never been able to talk about to anybody else. And I think that the fact that I didn't know this person and was never going to meet them again as long as I live... it wouldn't.... make any difference to them because they don't know me from Adam*.

*DP34w: There's Depression Alliance and there's Samaritans .. totally anonymous.. you need somebody that's not connected to you.. A complete stranger, that's what it has to be*.

Finally, two female respondents suggested that they found it difficult being the focus of attention and found a more distant and less personal relationship easier with their GP. DP18w described how she felt 'flustered' by the concern focused on her while DP11w described how she 'just wanted to blend into the background':-

*DP18w: I've always felt that she (GP) focused so much concern on me.. I got completely nervous and flustered. .. I didn't actually talk to her about anything really before my counsellor suggested that I should go and get diagnosed*.

*DP11w: I think my GP and my whole surgery were.. sometimes a little too supportive, it was almost a bit claustrophobic. Every time I went in there it's 'oh hello, how are you today?'... I just wanted to blend into the background because I felt I was down at the surgery so often I almost needed a chair in the waiting room with my name on it*.

### Differences in the accounts of men and women with depression

#### 1) DIFFERENT EMPHASIS IN COMMUNICATION (TALKING V LISTENING)

While both men and women valued the communication skills of health professionals, there were some gender differences in the emphasis put on different aspects of communication. For women, feeling that someone was really listening was particularly important:-

*DP22w: It was the first time somebody had sat and listened to me, listened to my concerns exclusively (counsellor)*.

*DP11w: They listened to me. It might have been complete rubbish what I was saying to them, but they did listen .. and they let me get angry and they let me say the things that I wanted to say (nurses and doctors in hospital)*.

*DP26w: I was listened to. I was heard. .. taken seriously (counsellor)*.

In contrast, men put more emphasis on the ways that health professionals enabled them to talk about their emotional distress. For example, DP03m discussed how his psychiatrist 'enabled' him to talk, DP01m discussed how he had moved from 'loathing' to talk about himself to being able to talk about anything to his psychiatrist and DP16m emphasised the importance of being able to 'unload and unburden':-

*DP03m: She (psychiatrist) was very good. She.. enabled me to talk*.

*DP01m: I got to the point where I would sit in a darkened room and not want to talk to anybody .. I explained all this to the psychiatrist.. which was a terrible ordeal because I loathed to talk about myself... One of his colleagues was the one that I saw for about 10 years.. he really helped a great deal.. cause I knew that I could talk to him about anything and everything*.

*DP16m: And you can just unload and unburden and that is so important. The unburdening in a controlled confidential environment.. particularly to someone who has experience and expertise and training, is very helpful (counsellor)*.

#### 2) MEN'S EMPHASIS ON GETTING PRACTICAL RESULTS FROM TALKING THERAPIES

Some respondents who had been initially doubtful about talking therapies were converted when they saw 'real' results. They contrasted practical, problem-solving therapies – often cognitive behavioural therapy (CBT) – with other forms of therapy which they saw as 'just talking'. The vast majority of respondents who constructed therapy in this way were men. For example, DP14m and DP13m rated their previous experience of therapy negatively (a 'talking shop', 'don't think any very practical thing came of it') in comparison with CBT which they felt had taught them to change their thinking. Other men found it frustrating when therapists were silent and would not answer direct questions, and preferred therapy which helped them to find solutions. For example, DP09m wanted 'practical help' and therapy which focused on the 'real world', while DP04m explained how he was initially extremely hostile to the idea of therapy, given his gender and class background, but then found it very helpful, especially when he saw it as 'practical problem solving' rather than as 'emoting':-

*DP09m: I don't think that this therapist (was).. really that helpful. Not helpful in practical terms because I actually needed practical help, I think, as well. Therapists don't say that... Systematic consultation. .tended to use a few cognitive techniques. I think they focused on the real world a lot more.. than some of the previous experiences*.

*DP04m: Therapy is anathema in my family. .we're not from that world.. that kind of class. It's not easy for blokes, especially not blokes from that kind of background...I think that blokes kind of like it (CBT).. (it's) not about emoting particularly. .though it's kind of good to sometimes tell someone something hurts you and to get it out.. I think if you can try and think about it as a practical problem solving thing rather than ...emotional masturbation.. then I think that will help*.

Although a number of women were also sceptical about talking therapies, only one woman (DP06w) framed cognitive therapy in a similar way to the men (i.e. as practical and effective as opposed to 'just talking'). DP06w was the nurse quoted above who found it difficult to talk to her GP about depression because she worked and socialised with him. In her narrative, she described how people referred to her as 'Wonder Woman' and said that 'I've always been one of these people that if anything needed to be done, I would do it because I might as well get on with it'. Therefore, it is possible she framed cognitive therapy in this 'practical' way due to her professional identity as a nurse and her personal identity as an efficient higher achiever who valued results:-

*DP06w: I have never really been a supporter of counselling .. I think you can talk about things till the cows come home and it doesn't actually make you any better. But this cognitive therapy, and the way that it looked at your behaviour.. seemed to me something that was worth trying. I think (it) was quite helpful because it did explore different areas that I hadn't given any thought to*.

## Discussion

In this paper, our aim was to examine the assumption that women are more articulate about depression while men are more silent, and to explore whether there are gender differences or similarities in the strategies men and women find useful when communicating with health professionals. We used a gender comparative approach to the data in order to explore commonalities among men and women, and diversity among women and among men, rather than focusing solely on gender differences. A number of studies [7-10,13] have found that people with depression find it difficult to find the vocabulary to discuss their problems, but our study is unusual in explicitly focusing on how men and women express emotional distress. Both men and women discussed how they had initially found it very difficult to articulate the experience of depression. However, in the interviews both men and women were able, and willing, to talk in detail about their feelings and experiences of depression to a male researcher. The expectation that men cannot (or will not) talk about depression was not borne out, challenging generalisations that men invariably demonstrate their strength through silence and stoicism (see also [45] for similar results).

Perhaps the most novel finding of our study was that, while many respondents valued personal relationships with health professionals, a minority found it easier to communicate with someone they didn't know ('talking to a stranger'). This finding applied to both men and women. It appeared that these respondents did not want to reveal their perceived weaknesses to a 'friend' because of pride or fear that it would change the relationship, and instead found it easier to discuss their problems with an anonymous stranger who remained separate from their day-to-day life. (This perhaps parallels Pollock's [18] assertion about patients suppressing emotional distress in order to 'save face' in the consultation). To our knowledge, this is a new finding in the literature. Existing research on GPs finds that they emphasise the importance of their communication skills [20,46] and their role as 'sympathetic listener' [47] in mental health consultations, while research on patients with depression emphasises their desire for continuity of care and to 'know' and 'be known by' their GP [14,15]. However, there are hints in the literature that some patients may feel differently. For example, Maxwell [15] notes in passing that two women in her study were reluctant to talk to their usual GPs about their emotional distress in case it influenced their GPs' perceptions of them; one did not want to be judged by her GP (who had seen her throughout her recent pregnancy) while the other perceived her GP to be a 'friend of the family'. Rogers and colleagues [10,13] make a more general point about the complexity of communication between patients and service providers. While patients valued GPs' communication skills, the authors suggest that 'there was ambiguity and unease expressed about the purpose of talk that went beyond what patients expected of the role of the doctor in a routine consultation' (p329). Some respondents believed that GPs were ill equipped to offer treatment such as talking therapies which went beyond a routine consultation because of their time constraints and their lack of detailed personal knowledge of patients' circumstances.

We identified a number of gender differences in our study. Women with depression were more likely than men to discuss the importance of finding health professionals who really listened to them. It is women rather than men who continue to do more 'emotional work' such as listening to people and giving emotional support in everyday relationships [48] so it could be that it is particularly valuable for women to feel 'heard' by health professionals and to talk without worrying about the emotional needs and responses of significant others.

Men in this study were more likely than women to talk about the value of therapy which had practical results. Interestingly, Good and Wood [49] found that men who held traditional, rigid gender roles measured by a gender role conflict scale (i.e. difficulty expressing emotions, uneasy about close relationships with other men) were more reluctant than other men to seek professional help for psychological problems. They suggest that one way to increase men's use of counselling services would be to focus less on emotional expressiveness and more on instrumental changes and control. Our work suggests that some men are already using this strategy to 're-frame' their experience of talking therapies.

This study has a number of limitations. First, our sample consisted of people with depression who were willing – and well enough – to volunteer to allow their accounts to be recorded for use on a website. However, respondents were given the option to conceal their identity on the website, and similar proportions (over half) of both men and women chose to do this. Secondly, it could be argued that the men in our sample were particularly unusual, given that depression is a condition commonly associated with women [50] and that culturally dominant constructions of masculinity emphasise emotional control and the denial of vulnerability [3,51]. Our previous work on the men in this sample [35] suggested that most men incorporated values associated with hegemonic masculinity into their narratives, and that only a minority consciously distanced themselves from culturally dominant forms of masculinity. We acknowledge that we are likely to have missed the truly 'strong and silent' depressed men [52] but it is difficult to see how we could have accessed and interviewed this group. Thirdly, it could also be argued that we are unlikely to have identified respondents when they are having difficulty in expressing their feelings about depression and communicating with health professionals. However, many respondents did discuss previous occasions when they felt they could not articulate their feelings. We do not claim numerical representation – this is not the purpose of maximum variation sampling – but we are confident that we have succeeded in eliciting a range of experiences that extend current understandings of depression.

## Conclusion

Our study suggests that the relationship between gender and engagement with health professionals among people with depression is complex. We found some gender similarities (both men and women found it difficult to recognise and articulate mental health problems and this had consequences for their ability to communicate with health professionals) and some gender differences (men tended to value skills which helped them to talk, while women valued listening skills in health professionals; respondents who emphasised the importance of getting practical results from talking therapies in their narratives, as opposed to other forms of therapy which they conceptualised as 'just talking', were almost exclusively men). We also found diversity among women and among men (some respondents valued a close personal relationship with health professionals, while others felt that this personal relationship was a barrier to communication and preferred 'talking to a stranger').

Our study has implications for health professionals. First, having a personal relationship with a GP can be both potentially advantageous (for those who value the more 'human' characteristics of health professionals) and problematic (for those who feel they cannot discuss their depression because of an existing friendship). General practitioners need to be sensitive to this and take their cue from patients, perhaps exploring whether they would find it helpful to discuss emotional issues with others in the practice if they have an ongoing personal or professional relationship with a patient. Secondly, GPs need to be sensitive to patients who have difficulties in expressing emotional distress. We found that this was a problem for both men and women, but that men in particular valued health professionals who helped them to talk about depression. Men with very traditional gender role attitudes may have particular difficulties in expressing emotional distress [53], may be more prone to serious suicidal thoughts [2] and more reluctant to seek help for psychological problems [49]. Our data suggest that asking sympathetic but focused questions may be more helpful than general questions such as 'how do you feel?' Australian researchers have recently tested a mental health prompt list in primary care which is designed to help men disclose emotional distress [54]. It would be useful to test this instrument in other contexts. Finally, it is important that GPs do not assume that all women can, and all men cannot, articulate their feelings and needs about emotional distress and mental health issues. These assumptions may have consequences for treatment; the findings of some studies suggest that primary care professionals treat male patients who seek help more seriously than female patients, because of the assumption that men only seek help (particularly for emotional distress) when they are really ill, whereas women consult with more trivial problems [55,56]. Kilmartin [53] suggests that health professionals should become aware of their own assumptions about men and mental health, and we would argue that this suggestion should be applied to assumptions about women too.

## Competing interests

The author(s) declare that they have no competing interests.

## Authors' contributions

SZ conceived the initial study and KH suggested the secondary analysis of the data. DR carried out the interviews, identified emerging themes and led analyses of other papers from the study. CE undertook the gender analysis of the data and drafted this paper. All authors helped to refine the analysis, contributed to the final version of the paper and approved the final manuscript.

## Pre-publication history

The pre-publication history for this paper can be accessed here:


